# Personality is predictive of burnout but not of work engagement: A one-year prospective cohort study

**DOI:** 10.1371/journal.pone.0339258

**Published:** 2026-01-07

**Authors:** Toshiki Fukuzaki, Noboru Iwata

**Affiliations:** 1 Department of Clinical Psychology, Graduate School of Medical Sciences, Tottori University, Yonago, Japan; 2 Psychosocial Epidemiology, Graduate School of Nursing, Dokkyo Medical University, Mibu, Japan; Dong-A University College of Business Administration, KOREA, REPUBLIC OF

## Abstract

This prospective cohort study aimed to longitudinally verify correlations between dimensions of the five-factor model of personality with work engagement and burnout. In the study, an online survey was conducted twice, with a one-year interval, targeting regular employees at a Japanese company (baseline survey: November–December 2022, follow-up survey: November–December 2023). Data from 500 individuals (299 men, 201 women) who responded to both surveys were used for analysis. Hierarchical multiple regression analysis was used. Baseline scores and demographic variables were adjusted. Personality did not significantly correlate with work engagement. However, among job resources, significant correlations were observed for extrinsic rewards (*β* = 0.15) and coworker support (*β* = 0.12). Meanwhile, significant correlations were noted for burnout with degree of job demands (*β *= 0.10), neuroticism (*β* = 0.08), and conscientiousness (*β* = −0.08). When implementing organizational measures focusing on worker personality, those for preventing burnout are more effective compared with those promoting work engagement.

## Introduction

Recent research on occupational mental health has focused on so-called positive mental health measures that create positive attitudes toward work among workers and prevent mental disorders, such as depression [1]. The job demands–resources (JD-R) model is an occupational stress model supporting the basis for such measures [2,3]. This model provides a theoretical framework for the idea that enhanced job resources engage workers through the motivational process. The core psychological concept of this process is work engagement (WE), which is defined as “positive, fulfilling, work-related state of mind that is characterized by vigor, dedication, and absorption” [2]. Improved WE is known to lead to good mental and physical health [4], outstanding job performance [5], and life satisfaction [6].

Additionally, the JD-R model includes the negative aspect of health impairment [2,3]. The health impairment process describes the burnout caused by increased job demands, resulting in negative health effects, such as depression. Burnout, the central psychological concept of health impairment, was originally proposed by Freudenberger [7] as a work-related negative psychological state. Later, Maslach and Jackson [8] defined it as follows: “Burnout is a syndrome of emotional exhaustion and cynicism that occurs frequently among individuals who do ‘people work’ of some kind.” They considered it to be a psychological concept specific to service professionals. At present, burnout has been reported to arise in various types of occupation [9]. Burnout state increases symptoms of depression [10,11] and causes negative occupational consequences, such as absences due to illness [12] and leaving one’s profession [13].

WE and burnout independently predict general happiness [14]. Therefore, to enable workers to work energetically and actively, elevate their job performance, and avoid health impairment or negative work-related effects, workplaces require measures for improving WE and preventing burnout. In other words, comfortable working environments must be established. These workplace environment factors are explained in the JD-R model as the variables of job demands and resources [2,3].

Further, the JD-R model accounts for workers’ personal factors, which are referred to as personal resources [15]. Personal resources involve positive self-evaluation and refer to an individual’s sense of being able to successfully control and impact their environment [16]. Typical concepts in personal resources include sense of self-efficacy, optimism, and resilience [17]. These personal resources are the strengths of individual workers and are thought to impact WE [15]. The impact of personal resources should be considered, in addition to the workplace environment factors of job demands and resources, when investigating workers’ psychological health.

One variable of personal resources is personality, which has been investigated with respect to the JD-R model [18,19]. Personality encompasses behavioral tendencies that can be observed relatively consistently regardless of the situation or point in time [20]. Of the various personality theories, the five-factor model (FFM) of personality is thought to explain the most marked aspects of personality [21]. Therefore, correlations between the dimensions of the FFM of personality and indices such as well-being and job performance have been frequently studied in workers [22–24].

The FFM of personality is composed of the five factors of neuroticism, extraversion, conscientiousness, agreeableness, and openness to experience [21]. According to studies on the relation of burnout with the FFM [25,26], burnout has a positive correlation with neuroticism and a negative correlation with the other four factors. As for WE [27,28], research has shown a negative correlation with neuroticism and a positive correlation with the other four factors. Specifically, workers with high WE are those who are emotionally stable (neuroticism); bright, active, and sociable (extraversion); sincere and systematic (conscientiousness); highly cooperative resulting in good interpersonal relationships (agreeableness); and have a wide range of interests and rich imagination (openness). Meanwhile, workers with the opposite characteristics tend to have increased burnout. These previous studies focusing on workers’ personalities emphasize the need to consider individual factors in the context of organizations and workplace environments, where numerous studies on burnout and WE have been conducted.

However, studies that mention relationships between personality and burnout or WE have major limitations. First, most of them rely on cross-sectional investigation findings. As personality is relatively stable and not affected by the environment [20], if it does correlate strongly with burnout or WE, personality as measured at a set time point may be interpreted to predict future burnout and WE. However, the workplace environment factors of job demands and resources must be measured simultaneously to investigate the impact of personality considering the level of impact of the two aforementioned factors. In the health impairment process, job demands exhibit a positive correlation with burnout [2,3]. In the motivational process, job resources exhibit a positive correlation with WE [2,3]. Therefore, the above correlations need to be longitudinally compared against correlations between the FFM dimensions with burnout and WE. However, no such investigation has been performed.

The second limitation is related to the tools for measuring burnout. Among several available scales [29,30], the most commonly used scale is the Maslach Burnout Inventory [31]. However, various issues have been cited, including problems regarding conceptualization [32], psychometric flaws [33], and low practicality [34]. To overcome these issues, Schaufeli et al. [9] combined deductive and inductive approaches to develop the Burnout Assessment Tool (BAT). It is considered to be fit for screening individuals or organizational populations. In this study, we used the BAT to measure burnout more accurately and to verify how it correlates with personality.

**Fig 1:**
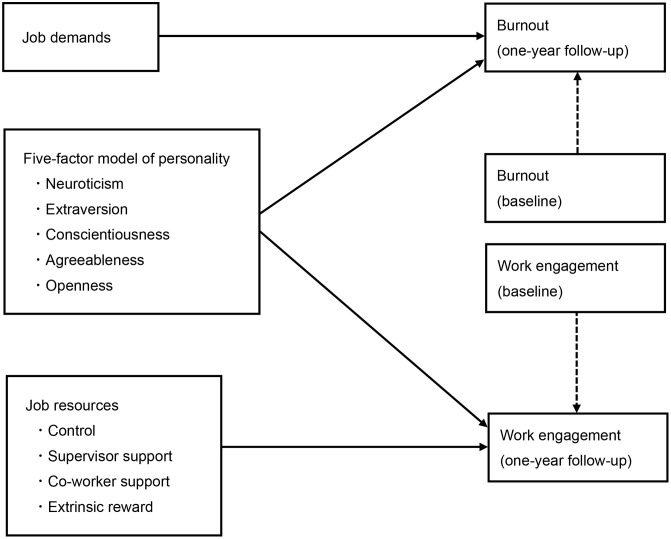
Conceptual model of the hypothesized longitudinal relationships among the variables of interest. *Note.* Solid arrows represent hypothesized longitudinal associations. Dashed arrows indicate baseline adjustment (i.e., controlling for work engagement and burnout at baseline).

Thus, we performed a prospective cohort study to longitudinally investigate correlations between worker personality and both WE and burnout. To account for the impact of workplace environment factors, we also measured the job demand and resources concepts of the JD-R model, and then compared correlations between personality and the intensity of job demands and resources (Fig 1).

## Materials and methods

### Participants

We conducted a one-year prospective cohort study online, using a panel of monitors registered with Rakuten Insight, Inc., a private online research company in Japan, with approximately 2.2 million registered monitors (1.3 and 0.9 million men and women, respectively). As the panel consisted of people with diverse attributes, as survey participants, we selected regular employees working in typical organizations and corporations. Non-regular employees, self-employed workers, freelancers, and similar were not included in the survey. Additionally, workers who resigned or whose employment changed to a non-regular type between the baseline and follow-up surveys were excluded from the follow-up survey. In other words, the survey targeted workers who were regularly employed at specific organizations at the baseline and those who continued to work at similar organizations as regular employees at the time of the follow-up survey. A baseline survey was conducted from late November to early December 2022, and valid responses were obtained from 1,500 workers. A year later, from late November to early December 2023, a follow-up survey was conducted, and valid responses were obtained from 500 individuals (299 men and 201 women) who had responded to both surveys (follow-up rate: 33.3%).

To examine potential selection biases, we compared the demographic characteristics and scores on psychology scales between participants who completed two surveys (Completers; *N* = 500) and those who did not take the follow-up survey (Dropouts; *N* = 1,000) (S1 Appendix). Differences were seen between Completers and Dropouts in terms of gender, educational background, and occupation. Regarding gender (χ^2^(1) = 26.14, *p* < 0.001), Completers (59.8%) comprised a higher proportion of men than Dropouts (45.8%). In terms of educational background (χ^2^(2) = 10.38, *p* < 0.01), Completers (64%) had a higher proportion of university and graduate school graduates than Dropouts (55.3%). Finally, regarding occupation (χ^2^(6) = 19.27, *p* < 0.01), Completers comprised a higher proportion of managers (Completers: 18.6%, Dropouts: 14.4%) and clerical support workers (Completers: 35.8%, Dropouts: 31.0%). No differences were seen in any of the scores for age, marital status, or psychological scales (all *n.s.*). In other words, compared with Dropouts, Completers comprised more men, more university and school graduates, and more managers and clerical support workers. Furthermore, Dropouts and Completers did not show significant differences regarding scores for any of the psychological scales.

This study was conducted with the approval of the Ethics Committee of Tottori University Faculty of Medicine (No. 22A110). All participants were provided with a comprehensive explanation of the study’s purpose and procedures, and gave their informed consent in written form through an online platform prior to participation. Minors were not included in this study.

## Measurement methods

### Demographic variables

We asked the participants about their gender, age, educational background, marital status, and occupation. For occupation, we used choices that complied with the International Labour Organization’s International Classification Standards [35]. We divided educational background into three groups: university/graduate school, vocational/junior college, and high school or below, and created a dummy variable, using high school and below as the reference. We divided marital status into three groups: single, married, and divorced/widowed, and formed a dummy variable, using divorced/widowed as the reference. We divided occupation into seven groups: managers, professionals, technicians and associate professionals, clerical support workers, service and sales workers, manual workers, and others. Manual workers included craft and related trades workers, plant and machine operators and assemblers, and workers in elementary occupations [35]. We created a dummy variable using “others” as the reference. These demographic variables were measured at baseline.

### Job demands

We used the job demand items from the Brief Job Stress Questionnaire [36], which comprise variables that correspond to the job demands included in the “job demand–control–support” model [37]. The questionnaire comprises six items pertaining to quantitative and qualitative workloads and has thus far been shown to have acceptable levels of internal consistency reliability and construct validity (quantitative workload, α = 0.77; qualitative workload, α = 0.68) [36]. Examples of questions about quantitative workload are “I have an extremely large amount of work to do” and “I can’t complete the work in the required time,” while examples of qualitative workload questions are “I have to pay very careful attention” and “My job is difficult as it requires a high level of knowledge and technical skill.” We asked the participants to answer these questions on a four-point scale, ranging from “1 = Very much so” to “4 = Not at all.” After making conversions to ensure that the higher the score, the greater the workload, we totaled the item scores and used the total as the job demand score (range = 6–24). Job demands were assessed solely at baseline.

### Job resources

As job resources, we measured control, supervisor and coworker support, and extrinsic reward. Control and supervisor and coworker support represent the variables for control and support in the “job demand–control–support” model [37]. For measuring these, we also used the items in the Brief Job Stress Questionnaire [36]. Control, supervisor support, and coworker support each comprised three items, and have thus far been shown to have acceptable levels of internal consistency, reliability, and construct validity (control, α = 0.65; supervisor support, α = 0.79; coworker support, α = 0.76) [36]. Examples of questions regarding control are “I can work at my own pace” and “I can choose how and in what order to do my work,” while examples of questions regarding supervisor and coworker support are “How freely can you talk with the following people?” and “How reliable are the following people when you are troubled?” The participants responded by assuming either a supervisor or their coworkers. We asked the participants to answer the questions regarding control on a four-point scale, ranging from “1 = Very much so” to “4 = Not at all,” and questions relating to supervisor and coworker support on a four-point scale, ranging from “1 = Extremely” to “4 = Not at all.” After making conversions to ensure that the higher the score, the greater the job discretion or the greater the support received from supervisors and coworkers, we totaled all the scores and used the totals as the scores for control, supervisor support, and coworker support (range of each variable = 3–12).

We measured extrinsic reward—a variable that corresponds to the reward in the effort–reward imbalance model [38]—using the abridged edition of the Japanese language model [39]. Extrinsic rewards comprise seven items and have thus far shown acceptable levels of internal consistency, reliability, and construct validity (α = 0.76) [39]. The scale comprises the subcategories of financial, esteem-related, and organizational rewards, with question examples being “Considering all my efforts and achievements, my salary/income is adequate,” “I receive the respect I deserve from my superior or a respective relevant person,” and “My job promotion prospects are poor.” We asked the participants to answer these questions on a four-point scale, ranging from “1 = Strongly disagree” to “4 = Strongly agree.” After making conversions to ensure that the higher the score, the greater the reward the participants received, we totaled all the scores and used the total as the score for extrinsic rewards (range = 7–28). Job resources were assessed solely at baseline.

### Work engagement

We used the abridged edition of the Japanese language Utrecht WE Scale [40]. WE comprises three factors—vigor, dedication, and absorption; each factor contains three items, resulting in a total of nine items. Examples of questions for vigor are “At my work, I feel bursting with energy” and “At my job, I feel strong and vigorous;” for dedication, they are “I am enthusiastic about my job” and “My job inspires me;” for absorption, they are “I feel happy when I am working intensely” and “I am immersed in my work.” We asked the participants to consider to what extent these questions applied to them and answer on a seven-point scale, ranging from “0 = Never” to “6 = Always/Every day.” We anticipated three subfactors for WE. However, with the Japanese language edition, a one-factor structure is appropriate and is shown to have favorable levels of internal consistency, reliability, and stability (α = 0.92), factor invariance, and construct validity [40]. Consequently, in this study, we treated the scale as having a one-factor structure and used the total score of all items as the WE score (range = 0–54). The higher the score, the higher the WE. WE was assessed at both baseline and follow-up.

### Burnout

We used the abridged edition of the Japanese language BAT [41]. The BAT comprises four core symptoms: exhaustion, mental distance, emotional impairment, and cognitive impairment; and two secondary symptoms: psychological distress and psychosomatic complaints. The Japanese edition is shown to have favorable levels of reliability (internal consistency and test–retest reliability), factor validity, and construct validity (core symptoms overall, α = 0.96; exhaustion, α = 0.93; mental distance, α = 0.86; emotional impairment, α = 0.91; cognitive impairment, α = 0.93; overall secondary symptoms, α = 0.92; psychological distress, α = 0.89; and psychosomatic distress, α = 0.87) [41]. For this study, we used the abridged edition that only measures the four core symptoms, each with three items. Examples of questions regarding exhaustion are “At work, I feel mentally exhausted” and “After a day at work, I find it hard to recover my energy;” for mental distance, they are “I struggle to find any enthusiasm for my work” and “I feel a strong aversion toward my job;” for emotional impairment, they are “At work, I feel unable to control my emotions” and “I do not recognize myself in how I react emotionally at work;” for cognitive impairment, they are “At work, I have trouble staying focused” and “When I’m working, I have trouble concentrating.” We asked the participants to consider to what extent these questions applied to them and answer on a five-point scale, ranging from “1 = Never” to “5 = Always.” Based on Sakakibara et al.’s [41] assertion that burnout measured via the Japanese BAT can be treated as a single dimension, we decided to treat burnout as having a one-factor structure, and treated the total scores of all items as the burnout score (range = 12–60). The higher the score, the higher the burnout level. Burnout was assessed at both baseline and follow-up.

### Personality

We used the abridged edition of the Big Five scale [42]—formulated by Wada [43] based on the Adjective Check List [44]—to measure personality in the FFM. It measures neuroticism with five items, extraversion with five items, conscientiousness with seven items, agreeableness with six items, and openness to experience with six items, for a total of 29 items. This scale is shown to have internal consistency reliability, factor structure validity, and convergent validity within the acceptable range (neuroticism, α = 0.79; extraversion = 0.84; conscientiousness = 0.75; agreeableness = 0.77; openness = 0.77). Questions relating to neuroticism covered “tendency to become anxious” and “worrywart;” extraversion covered “sociable” and “cheerful;” conscientiousness covered “methodical” and “meticulous;” agreeableness covered “gentle” and “generous;” and openness covered “original” and “versatile.” We asked the participants to consider to what extent these questions usually applied to them and answer on a five-point scale, ranging from “1 = Applies very much” to “5 = Does not apply at all.” After making conversions to ensure that the higher the score, the stronger the specific personality traits, we totaled each score and used it as the personality trait score (neuroticism and extraversion range = 5–25, agreeableness and openness range = 6–30, and conscientiousness range = 7–35). Personality traits were assessed only at baseline.

### Statistical analysis

We calculated the Pearson correlation coefficients between demographic variables and scores for each scale. To confirm the internal consistency and reliability of the psychological scales, we calculated the McDonald’s omega (ω) coefficient.

We conducted a hierarchical multiple regression analysis, using the WE score at the follow-up as the response variable, and entered explanatory variables in the following order. First, to control the influence of WE at the baseline, we entered the baseline WE scores (Step 1). Next, we entered the demographic variables of gender, age, educational background (a dummy variable using high school or below as the reference), marital status (a dummy variable using divorced or widowed as the reference), and occupation (a dummy variable using others as the reference) (Step 2). We then entered job demands (Step 3), followed by job resources (control, supervisor and coworker support, and extrinsic rewards) (Step 4). Finally, we entered each personality variable (neuroticism, extraversion, conscientiousness, agreeableness, and openness) (Step 5). Additionally, we conducted a hierarchical multiple regression analysis, using the burnout scores at the follow-up as the response variable and following the same order as in the case of WE. We then compared the results for WE and burnout.

To ensure the validity and robustness of the regression analyses, several diagnostic and sensitivity procedures were conducted. First, inverse probability weighting was applied as a sensitivity analysis to account for potential attrition bias, and results were compared with the unweighted model. Second, regression diagnostics were performed to examine model assumptions, including linearity, normality, homoscedasticity, multicollinearity, and influential observations using standard statistical tests and diagnostic plots (e.g., residuals vs fitted, Q–Q, scale–location, and residuals vs leverage plots). The Durbin–Watson statistic was used to test autocorrelation, and variance inflation factors (VIFs) were computed to evaluate multicollinearity. Third, 95% percentile bootstrap confidence intervals (5,000 resamples) were computed to assess the stability of the regression coefficients. Finally, the Breusch–Pagan test was used to assess heteroscedasticity, and HC3-robust standard errors were applied when heteroscedasticity was detected.

For all analyses, the significance level was set at 5%, and statistical analyses were performed using R version 4.3.2.

## Results

Table 1 shows the analysis of participant demographic characteristics and scale scores. Their mean age was 45.9 years (SD = 11.9), and approximately 60% were men. Regarding their educational background, most (64.0%) were university and graduate school graduates; 56.6% were married. The leading occupation was clerical support workers (35.8%), followed by professionals (20.8%) and managers (18.6%).

**Table 1:** Demographic characteristics and scale scores (*N* = 500).

Demographic characteristics	Mean (SD)	*n* (%)
Age (years)	45.9 (11.9)	
Gender		
Men		299 (59.8)
Women		201 (40.2)
Education		
University/graduate school graduate		320 (64.0)
Vocational school/college graduate		102 (20.4)
High school graduate or lower		78 (15.6)
Marital status		
Unmarried		171 (34.2)
Married		283 (56.6)
Divorce or bereavement		46 (9.2)
Occupations		
Manager		93 (18.6)
Professional		104 (20.8)
Technicians and associate professional		40 (8.0)
Clerical support worker		179 (35.8)
Service and sales worker		34 (6.8)
Manual worker^a^		21 (4.2)
Others		29 (5.8)
**Scale scores**	**Mean (SD)**	***n* (%)**
Five-factor model		
Neuroticism	16.0 (3.7)	
Extraversion	15.2 (3.7)	
Conscientiousness	22.5 (4.3)	
Agreeableness	19.2 (3.6)	
Openness	18.3 (3.8)	
Job demands	16.3 (3.9)	
Job resources		
Control	8.0 (2.1)	
Supervisor support	7.4 (2.3)	
Co-worker support	7.8 (2.2)	
Extrinsic reward	18.0 (3.5)	
Work engagement		
Baseline	21.5 (12.3)	
One-year follow-up	20.1 (12.5)	
Burnout		
Baseline	31.7 (8.9)	
One-year follow-up	31.5 (9.5)	

^a^Manual worker include craft and related trades worker, plant and machine operator and assembler, and elementary occupation.

Paired-sample t-tests were conducted to examine changes in WE and burnout over one year, showing that WE significantly decreased (baseline: Mean = 21.5, SD = 12.3; follow-up: Mean = 20.1, SD = 12.5, *t*(499) = −2.88, *p* < 0.01) while burnout remained unchanged (baseline: Mean = 31.7, SD = 8.9; follow-up: Mean = 31.5, SD = 9.5, *t*(499) = −0.55, *n.s.*).

Table 2 shows the Pearson correlation and ω coefficients. Each personality variable showed a weak but significant correlation with WE scores at the follow-up (neuroticism: negative correlation; others: positive correlation). The results were similar to the burnout scores at the follow-up (neuroticism: positive correlation; others: negative correlation). All ω coefficients exceeded 0.80, and all the scales used in this study showed favorable internal consistency.

**Table 2:** Pearson’s correlation coefficients and reliability estimates (McDonald’s omega) (*N* = 500).

		1	2	3	4	5	6	7	8	9	10	11	12	13
1	Gender^a^	–												
2	Age	0.020	–											
3	University/graduate school graduate^b^	−0.286***	−0.095*	–										
4	Vocational school/college graduate^b^	0.263***	0.020	−0.675***	–									
5	Unmarried^c^	0.166***	−0.396***	0.014	0.033	–								
6	Married^c^	−0.245***	0.284***	0.066	−0.087	−0.823***	–							
7	Manager^d^	−0.203***	0.374***	0.134**	−0.076	−0.204***	0.170***	–						
8	Professional^d^	0.052	0.034	0.056	0.010	−0.120**	0.091*	−0.245***	–					
9	Technicians and associate professional^d^	−0.197**	−0.095*	0.083	−0.076	0.067	−0.039	−0.141**	−0.151**	–				
10	Clerical support worker^d^	0.324***	−0.197***	−0.031	−0.026	0.236***	−0.205***	−0.357***	−0.383***	−0.220***	–			
11	Service and sales worker^d^	−0.027	−0.043	−0.128**	0.100*	0.056	−0.020	−0.129**	−0.138**	−0.080	−0.202***	–		
12	Manual worker^d^	−0.111*	0.004	−0.134**	0.042	−0.067	0.043	−0.100*	−0.107*	−0.062	−0.156***	−0.057	–	
13	JD*	−0.029	−0.045	0.063	0.016	−0.067	0.067	0.024	0.227***	−0.004	−0.220***	0.030	−0.020	(0.91)
14	Control	−0.082	0.108*	−0.037	0.019	−0.135**	0.119**	0.213***	−0.019	0.024	−0.130**	−0.071	−0.025	0.026
15	SS^f^	−0.066	−0.024	0.009	−0.003	−0.061	0.064	0.042	0.014	0.011	−0.053	−0.011	−0.016	−0.015
16	CS^g^	−0.004	−0.015	−0.021	0.003	−0.075	0.076	0.044	0.033	−0.035	−0.102*	0.023	0.023	0.017
17	ER^h^	−0.018	−0.002	−0.012	0.020	−0.065	0.068	0.148**	0.007	0.052	−0.112*	−0.036	−0.032	−0.048
18	N^i^	0.059	−0.187***	−0.003	0.021	0.186***	−0.177***	−0.137**	0.008	−0.002	0.023	0.067	0.003	0.139**
19	E^i^	0.009	0.023	−0.061	0.013	−0.148**	0.134**	0.074	0.038	−0.003	−0.101*	−0.025	0.019	0.055
20	C^k^	0.139**	0.217***	−0.010	0.021	−0.042	−0.020	0.182***	−0.024	−0.089*	0.006	−0.081	−0.022	0.093*
21	A^l^	−0.027	0.150**	0.021	−0.022	−0.102*	0.092*	0.105*	−0.004	−0.061	−0.016	−0.046	−0.053	0.018
22	O^m^	−0.103*	0.035	0.070	−0.066	−0.078	0.090*	0.113*	0.053	0.010	−0.124**	−0.065	0.007	0.065
23	WE at baseline^n^	0.021	0.194***	−0.001	0.008	−0.128**	0.102*	0.197***	0.062	0.010	−0.145**	−0.105*	−0.024	0.088*
24	WE at one-year follow-up^n^	0.024	0.274***	0.025	0.023	−0.089*	0.034	0.227***	0.028	−0.001	−0.105*	−0.103*	−0.079	0.073
25	BO at baseline^o^	−0.011	−0.192***	0.005	0.035	0.098*	−0.087	−0.146**	0.025	−0.029	0.019	0.068	0.045	0.288***
26	BO at one-year follow-up^n^	−0.047	−0.196***	0.015	0.011	0.105*	−0.082	−0.128**	0.001	0.022	0.010	0.066	0.039	0.263***
		**14**	**15**	**16**	**17**	**18**	**19**	**20**	**21**	**22**	**23**	**24**	**25**	**26**
14	Control	(0.81)												
15	SS^f^	0.303***	(0.90)											
16	CS^g^	0.289***	0.748***	(0.88)										
17	ER^h^	0.382***	0.437***	0.369***	(0.87)									
18	N^i^	−0.203***	−0.176***	−0.178***	−0.248***	(0.87)								
19	E^j^	0.224***	0.250***	0.344***	0.247***	−0.226***	(0.88)							
20	C^k^	0.179***	−0.001	0.014	0.128**	−0.245***	−0.010	(0.87)						
21	A^l^	0.187***	0.079	0.122**	0.131**	−0.296***	0.268***	0.357***	(0.87)					
22	O^m^	0.246***	0.152**	0.165***	0.152**	−0.099*	0.477***	0.169***	0.318***	(0.87)				
23	WE at baseline^n^	0.288***	0.316***	0.295***	0.386***	−0.261***	0.300***	0.287***	0.237***	0.271***	(0.98)			
24	WE at one-year follow-up^n^	0.269***	0.258***	0.277***	0.376***	−0.199***	0.200***	0.245***	0.203***	0.210***	0.653***	(0.98)		
25	BO at baseline^o^	−0.363***	−0.322***	−0.321***	−0.434***	0.354***	−0.264***	−0.243***	−0.308***	−0.254***	−0.338***	−0.355***	(0.96)	
26	BO at one-year follow-up^o^	−0.279***	−0.243***	−0.232***	−0.330***	0.354***	−0.222***	−0.260***	−0.287***	−0.200***	−0.335***	−0.387***	0.709***	(0.96)

*p < 0.05; **p < 0.01; ***p < 0.001.

^a^Men = 0,Women = 1.

^b^High school graduate or lower group is a reference.

^c^Divorce or bereavement group is a reference.

^d^Others group is a reference.

^e^JD = job demands.

^f^SS = supervisor support.

^g^CS = co-worker support.

^h^ER = extrinsic reward.

^i^N = neuroticism.

^j^E = extraversion.

^k^C = conscientiousness.

^l^A = agreeableness.

^m^O = openness.

^n^WE =work engagement.

^o^BO =burnout.

Tables 3 and 4 show the results of hierarchical multiple regression analyses of WE and burnout, respectively. With WE, the coefficient of determination increased significantly in Step 2 (Δ*R*^2 ^= 0.039, Δ*F*(12, 486) = 2.958, *p* < 0.001), in which demographic variables were entered, and age showed a significant positive correlation (*β* = 0.164, *t *= 4.133, SE = 0.040, *p* < 0.001). Fur*t*hermore, R² increased significantly in Step 4 (Δ*R*^*2*^ = 0.031, Δ*F*(4, 481) = 7.340, *p* < 0.001), where job resource variables were introduced. Among these, extrinsic reward (*β *= 0.148, *t *= 3.767, SE = 0.039, *p* < 0.001) and coworker support (*β* = 0.121, *t* = 2.410, SE = 0.050, *p* < 0.05) showed significan*t* positive associations wi*t*h WE. No significant changes were seen in the coefficient of determination between Step 5, in which personality variables were entered, and Step 3, in which job demands were entered. In Step 5, which included all variables, the most strongly associated significant explanatory variables, excluding baseline WE score and the demographic variable of age, were extrinsic rewards (*β* = 0.153, *t *= 3.822, SE = 0.040, *p* < 0.001), followed by coworker support (*β *= 0.129, *t *= 2.510, SE = 0.051, *p* < 0.05).

**Table 3:** Association of work engagement at baseline, demographic characteristics, job demands, job resources, and personality with work engagement at one-year follow-up: hierarchical multiple regression analysis (*N* = 500).

	Step 1		Step 2		Step 3		Step 4		Step 5	
	β	95%CI	β	95%CI	β	95%CI	β	95%CI	β	95%CI
Work engagement at baseline	0.653	0.587–0.720	0.618	0.550–0.686	0.616	0.548–0.684	0.537	0.463–0.610	0.535	0.458–0.613
Demographic characteristics										
Gender^a^			-0.016	-0.173–0.141	-0.018	-0.176–0.139	-0.037	-0.192–0.117	-0.031	-0.189–0.127
Age (years)			0.164	0.086–0.242	0.168	0.089–0.246	0.199	0.121–0.276	0.196	0.117–0.275
Education (reference = high school graduate or lower)										
University/graduate school graduate			0.165	-0.029–0.359	0.159	-0.035–0.353	0.183	-0.007–0.373	0.174	-0.018–0.365
Vocational school/college graduate			0.175	-0.048–0.398	0.170	-0.053–0.394	0.180	-0.038–0.398	0.179	-0.040–0.398
Marital status (reference = divorce or bereavement)										
Unmarried			-0.058	-0.315–0.200	-0.055	-0.313–0.202	-0.044	-0.295–0.207	-0.054	-0.307–0.199
Married			-0.205	-0.443–0.033	-0.206	-0.444–0.032	-0.229	-0.461–0.003	-0.228	-0.462–0.006
Occupation (reference = others)										
Manager			0.109	-0.220–0.439	0.106	-0.224–0.436	0.057	-0.268–0.381	0.066	-0.262–0.394
Professional			-0.020	-0.333–0.293	-0.033	-0.347–0.282	-0.030	-0.338–0.277	-0.023	-0.333–0.288
Technicians and associate professional			0.018	-0.342–0.379	0.019	-0.342–0.380	0.016	-0.339–0.370	0.031	-0.327–0.389
Clerical support worker			0.020	-0.281–0.321	0.029	-0.273–0.331	0.072	-0.224–0.369	0.082	-0.218–0.381
Service and sales worker			-0.096	-0.467–0.275	-0.101	-0.472–0.271	-0.107	-0.469–0.256	-0.095	-0.460–0.270
Manual worker			-0.257	-0.678–0.163	-0.256	-0.677–0.164	-0.244	-0.654–0.167	-0.233	-0.647–0.181
Job demands					0.030	-0.038–0.099	0.047	-0.020–0.114	0.045	-0.024–0.114
Job resources										
Control							0.027	-0.045–0.099	0.023	-0.051–0.097
Supervisor support							-0.065	-0.166–0.036	-0.067	-0.168–0.035
Co-worker support							0.121	0.022–0.219	0.129	0.028–0.230
Extrinsic reward							0.148	0.071–0.226	0.153	0.074–0.231
Personality										
Neuroticism									0.013	-0.061–0.086
Extraversion									-0.038	-0.119–0.044
Conscientiousness									0.002	-0.075–0.078
Agreeableness									0.014	-0.060–0.089
Openness									0.036	-0.042–0.114
R^2^	0.427		0.466		0.467		0.497		0.499	
Adjusted R^2^	0.426***		0.452***		0.451***		0.479***		0.475***	
Δ**R**^2^			0.039***		0.001		0.031***		0.002	

*p < 0.05; **p < 0.01; ***p < 0.001

^a^Man = 0, Woman = 1

**Table 4:** Association of burnout at baseline, demographic characteristics, job demands, job resources, and personality with burnout at one-year follow-up: hierarchical multiple regression analysis (*N* = 500).

	Step 1		Step 2		Step 3		Step 4		Step 5	
	*β*	95%CI	*β*	95%CI	*β*	95%CI	*β*	95%CI	*β*	95%CI
Burnout at baseline	0.709	0.647–0.771	0.697	0.632–0.761	0.675	0.608–0.742	0.649	0.570–0.727	0.596	0.513–0.678
Demographic characteristics										
Gender^a^			-0.071	-0.221–0.079	-0.079	-0.229–0.071	-0.084	-0.236–0.067	-0.074	-0.227–0.079
Age (years)			-0.050	-0.125–0.025	-0.044	-0.119–0.031	-0.051	-0.127–0.025	-0.032	-0.108–0.044
Education (reference = high school graduate or lower)										
University/graduate school graduate			-0.019	-0.205–0.167	-0.031	-0.217–0.154	-0.037	-0.224–0.149	-0.034	-0.219–0.151
Vocational school/college graduate			-0.023	-0.237–0.190	-0.033	-0.246–0.180	-0.029	-0.243–0.185	-0.025	-0.237–0.186
Marital status (reference = divorce or bereavement)										
Unmarried			0.056	-0.190–0.303	0.062	-0.183–0.308	0.056	-0.190–0.303	0.031	-0.213–0.275
Married			0.018	-0.210–0.246	0.012	-0.215–0.239	0.014	-0.214–0.241	0.009	-0.217–0.235
Occupation (reference = others)										
Manager			0.004	0.312–0.321	-0.015	-0.331–0.301	0.002	-0.317–0.321	0.038	-0.279–0.356
Professional			0.006	-0.295–0.306	-0.031	-0.332–0.270	-0.032	-0.335–0.271	-0.026	-0.328–0.275
Technicians and associate professional			0.123	-0.224–0.470	0.114	-0.231–0.460	0.120	-0.229–0.469	0.113	-0.235–0.460
Clerical support worker			0.016	-0.273–0.305	0.034	-0.255–0.322	0.028	-0.264–0.320	0.056	-0.234–0.346
Service and sales worker			0.078	-0.277–0.433	0.068	-0.286–0.422	0.059	-0.297–0.414	0.046	-0.307–0.398
Manual worker			0.046	-0.357–0.450	0.049	-0.353–0.450	0.040	-0.363–0.443	0.056	-0.344–0.457
Job demands					0.075	0.007–0.143	0.080	0.011–0.150	0.096	0.026–0.167
Job resources										
Control							-0.025	-0.098–0.047	-0.011	-0.083–0.061
Supervisor support							-0.026	-0.125–0.073	-0.038	-0.136–0.060
Co-worker support							0.014	-0.083–0.111	0.028	-0.070–0.126
Extrinsic reward							-0.027	-0.104–0.050	-0.013	-0.089–0.063
Personality										
Neuroticism									0.082	0.011–0.153
Extraversion									-0.033	-0.112–0.045
Conscientiousness									-0.075	-0.149–-0.002
Agreeableness									-0.035	-0.107–0.038
Openness									-0.001	-0.076–0.075
***R*** ^**2**^	0.502		0.509		0.514		0.516		0.533	
**Adjusted *R*** ^**2**^	0.501***		0.496***		0.500***		0.498***		0.510***	
***ΔR*** ^**2**^			0.007		0.005*		0.002		0.017**	

*p < 0.05; **p < 0.01; ***p < 0.001

^a^Man = 0, Woman = 1

As shown in S2 Appendix, the inverse probability weights-weighted regression yielded results consistent with those of the unweighted model (Table 3), indicating robustness to potential attrition bias. Baseline WE, age, coworker support, and extrinsic reward remained significant (i.e., their 95% confidence intervals did not include zero) predictors of WE at the one-year follow-up.

The Durbin–Watson statistic was 1.949, with no significant autocorrelation in residuals. Multicollinearity was acceptable, with VIF values in the range of 1.174–5.093 (all tolerance ≥ 0.196). The model’s root mean square error (RMSE) was 0.707, indicating adequate fit and predictive accuracy. Diagnostic plots (residuals vs. fitted, Q–Q, scale–location, and residuals vs. leverage plots) indicated no substantial deviations from linearity, normality, or homoscedasticity, and no influential observations were detected (Cook’s distance < 1). Cook’s distances were in the range of 0.000–0.074, with none exceeding the conventional cut-off of 1.0, indicating the absence of influential outliers. To assess regression coefficient robustness, 95% percentile bootstrap confidence intervals (5,000 resamples) were computed. The bootstrap-estimated coefficients closely matched the original standardized regression coefficients, suggesting findings’ stability and that they were not driven by sampling variability. The Breusch–Pagan test indicated heteroscedasticity (BP = 61.566, df = 23, *p* < 0.001). Therefore, when estimating standardized regression coefficients, HC3-robust standard errors were used to adjust for heteroscedasticity. Overall, these diagnostic results suggest that the regression model met the key assumptions and provided robust and stable estimates.

With burnout, the coefficient of determination increased significantly in Step 3 (Δ*R*^2 ^= 0.005, Δ*F*(1, 485) = 4.698, *p* < 0.05), in which job demand variables were entered, and showed a significant positive correlation (*β *= 0.075, *t *= 2.167, SE = 0.035, *p* < 0.05). Moreover, *t*he coefficient of determination increased significantly in Step 5 (Δ*R*^2 ^= 0.017, Δ*F*(5, 476) = 3.414, *p* < 0.01), in which personality variables were entered; neuroticism showed a positive (*β* = 0.082, *t *= 2.255, SE = 0.036, *p* < 0.05) and conscientiousness a negative correla*t*ion (*β* = −0.075, *t *= −2.016, SE = 0.037, *p* < 0.05). No significant changes were seen in the coefficient of de*t*ermination between Step 2, in which demographic variables were entered, and Step 4, in which job resources were entered. In Step 5, in which all significant explanatory variables excluding burnout scores at the baseline were entered, job demands showed the strongest correlation (*β* = 0.096, *t *= 2.678, SE = 0.036, *p* < 0.01), followed by neuroticism (*β *= 0.082, *t *= 2.255, SE = 0.036, *p* < 0.05) and conscientiousness (*β* = −0.075, *t *= −2.016, SE = 0.037, *p* < 0.05), which were a*t* about the same level.

As shown in S3 Appendix, the inverse probability weights-weighted regression yielded results consistent with those of the unweighted model (Table 4). Baseline burnout, job demands, neuroticism, and conscientiousness remained significant (i.e., the 95% confidence intervals for these predictors did not include zero) predictors of burnout at follow-up.

The Durbin–Watson statistic was 1.904, with no significant autocorrelation seen in residuals. For the burnout model, multicollinearity was acceptable, with VIF values in the range of 1.313–5.113 (all tolerance ≥ 0.196). The model’s RMSE was 0.683, indicating adequate fit and predictive accuracy. Diagnostic plots (residuals vs. fitted, Q–Q, scale–location, and residuals vs. leverage plots) indicated no substantial deviations from linearity, normality, or homoscedasticity, and no influential observations were detected (Cook’s distance < 1). Cook’s distances were in the range of 0.000–0.031, with none exceeding the conventional cut-off of 1.0, indicating the absence of influential outliers. To assess regression coefficient robustness, 95% bootstrap confidence intervals (5,000 resamples) were computed. The bootstrap estimates closely matched the original standardized regression coefficients, suggesting findings’ stability and that they were not driven by sampling variability. The Breusch–Pagan test indicated heteroscedasticity (BP = 48.139, df = 23, *p* < 0.01). Therefore, HC3-robust standard errors were used to adjust for heteroscedasticity when estimating standardized regression coefficients. Overall, these diagnostic outcomes suggest that the regression model met key assumptions and provided robust and stable estimates.

## Discussion

The objective of this prospective cohort study was to verify how worker personality correlates with WE and burnout. Our results revealed that WE did not significantly correlate with personality but correlated with extrinsic rewards and coworker support. Meanwhile, burnout correlated significantly with the two personality factors of neuroticism and conscientiousness as well as with job demands.

Fukuzaki and Iwata [45] compared correlations among affectivity traits with WE and psychological distress and found that WE is less impacted by affectivity traits compared with psychological distress. Our results support this finding. Thus, when focusing on worker personality, measures for preventing burnout are more effective compared with measures for promoting WE. Furthermore, our results for burnout showed no significant correlations for job resource variables in Steps 4 or 5. These support the findings of Bianchi [46] that indicated that correlations with personality traits are underestimated in burnout research. Notably, our results differed from those of research claiming that rich job resources prevent falling into a state of burnout [31,47,48]. What this means is that our findings reinforce the importance of focusing on personality traits, rather than job resources, in developing countermeasures for burnout.

Regarding the FFM of personality, our results showed a correlation between burnout and both neuroticism and conscientiousness, indicating that these two factors are more valid than the other factors as key markers for predicting burnout. Neuroticism has been previously shown to correlate more strongly with burnout than the other factors [25,26,46] and seems to function as a vulnerability factor that makes burnout more likely. Heightened neuroticism is linked to vulnerability to a series of negative emotions that comprise depression, anxiety, and anger [49], making it likely that the individual will exhibit maladaptation [50]. Given that heightened neuroticism is also related to difficulty experiencing job satisfaction [51], our results appear to support the findings of past studies.

We found that conscientiousness correlated with burnout to the same extent as neuroticism. This indicates that conscientiousness is an important marker and a protective factor for burnout to the same extent as neuroticism is a risk factor. Conscientiousness is a personality trait that is linked to stronger diligence, deliberateness, and autonomous ability. It is known to correlate with the job-related markers of performance and income [22,52]. Cross-sectional reports have shown that conscientiousness correlates more strongly with WE than other factors [28,53], whereas data from our longitudinal study indicate that conscientiousness correlates significantly with burnout but not with WE. Greater conscientiousness has been shown to be linked to health behaviors for maintaining good lifestyle habits [54]. As such, highly conscientious workers may react to job stressors by deliberately and effectively engaging in stress coping methods in their free time and days off.

Meanwhile, our results showed that WE did not exhibit a significant correlation with personality. This signifies that personality does not impact WE to the extent that job resources do. Organizational interventions to enhance WE should therefore prioritize the sufficient provision of job resources, such that work environments become propitious for employees to develop perceptions of a supportive and comfortable workplace. Greater WE means that workers become absorbed in work and find joy from it. When this happens, they gain more job resources than before and become more engaged in their work. This process is called a gain spiral [55,56]. Workers with a personality profile that facilitates increased WE may be more likely to enter this gain process. Once in a gain process, they are able to acquire more job resources and benefit from increased WE. Therefore, the impact of personality on WE may gradually decrease.

We also found that job demands correlated with burnout and job resources correlated with WE, which supports the JD-R model [2,3,57]. However, of the various job resources, extrinsic rewards most strongly correlated with WE, followed by coworker supports. Oshio et al. [58] used a fixed-effect model on data from a Japanese occupation cohort study and investigated correlations between job resources and WE. They emphasized the strength of the correlation between extrinsic rewards with WE out of the various job resource variables. We also found similar results. When enhancing job resources as an organizational measure to promote better WE, extrinsic rewards appear to be an essential variable.

Meanwhile, in contrast to past studies [5,12,59], our results indicated that coworker supports significantly correlated with WE whereas control and supervisor supports did not. This may be explained by the fact that in Japan, receiving generous support from superiors causes subordinates to feel apologetic. As such, the context of Japan may explain the weaker correlation between support from superiors and WE compared with other countries [60]. Moreover, one study reported that if the baseline WE is adjusted, correlations with support from superiors and colleagues as well as extrinsic rewards disappear while control remains [61]. Taking our results into account, improving WE among workers in Japan means paying more attention to opportunities for workplace interaction with colleagues rather than support from superiors or work responsibility.

Our results demonstrated that assessing workers’ personalities and leveraging such data would be an effective measure for preventing burnout. For example, when industrial health staff offer training or information to help employees improve their self-care skills, such training may be more effective if the content covers items related to stress management, considering stressor susceptibility in workers with strong neuroticism. Moreover, managers should place workers who have low conscientiousness, short work experience, and difficulty working systematically in posts that receive increased support from colleagues and that do not involve too much multitasking. Skillful leveraging of employee personality findings by industrial health staff and management could prevent mental health problems and help cultivate more talented human resources.

The present study had several limitations. First, our sample consisted of monitors registered at the same online research company. That is, participants were recruited using non-probability sampling through an online survey panel, potentially limiting sample representativeness of the general Japanese workforce. Therefore, the findings should be interpreted with caution. In addition, although we conducted two surveys and found no differences in the scale scores between dropouts and respondents targeted for analysis, we observed significant differences in the demographic items of gender, highest academic achievement, and occupational type (S1 Appendix). Thus, our results might have been impacted by selection bias. Second, although personality and WE were not found to be significantly correlated in this study, WE, which is composed of positive items, is known to easily reflect cultural differences [62]. Furthermore, the present study did not include macro variable measures such as collectivism or organizational culture, nor did it examine their relationships with WE, burnout, or personality. Therefore, our results may not be extrapolated to apply to other countries, and similar investigations need to be conducted outside of Japan. Third, job demands and resources in the JD-R model comprise multiple variables [2,3]. We only used some of them in this study. As such, verifications need to be performed using other variables. Fourth, this study found a significant decline in WE between baseline and follow-up, but we do not know the reasoning for this finding. Job resources may play an important role in maintaining and enhancing WE according to our evidence, but changes in these resources were not examined. To gain a more comprehensive understanding of WE fluctuations, future research should include repeated assessments of work-related factors such as job demands and resources. Finally, the hierarchical multiple regression analysis performed in this study set WE and burnout as response variables and job demands and resources as explanatory variables. Although we set the variables in accordance with the conventional JD-R model [2,3], reverse causal relations, such as those described in the Discussion, are possible. For example, high WE could result in the acquisition of more job resources, or falling into a state of burnout could result in job demands becoming greater than they previously were [57]. As the present study did not investigate these relationships, they need to be verified in future longitudinal research.

## Conclusions

Although burnout significantly correlated with job demands and the factors of neuroticism and conscientiousness, WE did not correlate with personality. Rather, WE correlated significantly with the job resources of extrinsic rewards and coworker support. Thus, preventing burnout may hinge on implementing management strategies that harness employees’ personality traits, whereas enhancing WE may require cultivating a resource-rich work environment that enables them to work effectively and comfortably.

## Supporting information

S1 Appendix Comparison of demographic characteristics and scale scores between survey completers and dropouts. ^a^Manual worker include craft and related trades worker, plant and machine operator and assembler, and elementary occupation. ^b^t-test. ^c^chi-square test. (DOCX)

S2 Appendix Association of work engagement at baseline, demographic characteristics, job demands, job resources, and personality with work engagement at one-year follow-up: hierarchical multiple regression analysis weighted by stabilized inverse probability weights (*N* = 500). *Note.* Weighted by stabilized inverse probability weights to adjust for potential attrition bias. Results correspond to the final step (Step 5) of the main analysis (Table 3). ^a^Man = 0, Woman = 1.
(DOCX)

S3 Appendix Association of burnout at baseline, demographic characteristics, job demands, job resources, and personality with burnout at one-year follow-up: hierarchical multiple regression analysis weighted by stabilized inverse probability weights (*N *= 500). *Note.* Weighted by stabilized inverse probability weights to adjust for potential attrition bias. Results correspond to the final step (Step 5) of the main analysis (Table 4). ^a^Man = 0, Woman = 1.
(DOCX)
