# Correction: Personality is predictive of burnout but not of work engagement: A one-year prospective cohort study

**DOI:** 10.1371/journal.pone.0342818

**Published:** 2026-02-12

**Authors:** Toshiki Fukuzaki, Noboru Iwata

In the Abstract section, there is an error in the seventh sentence. The correct sentence is: However, among job resources, significant correlations were observed for extrinsic rewards (*β* = 0.15) and coworker support (*β* = 0.13).

Table 2 was uploaded incorrectly. Please see the correct Table 2 here.

**Table 2:** Pearson’s correlation coefficients and reliability estimates (McDonald’s omega) (*N* = 500).

		1	2	3	4	5	6	7	8	9	10	11	12	13
1	Gender^a^	–												
2	Age	0.020	–											
3	University/graduate school graduate^b^	-0.286***	-0.095**	–										
4	Vocational school/college graduate^b^	0.263***	0.020	-0.675***	–									
5	Unmarried^c^	0.166***	-0.396***	0.014	0.033	–								
6	Married^c^	-0.245***	0.284***	0.066	-0.087	-0.823***	0.170***	–						
7	Manager^d^	-0.203***	0.374***	0.134**	-0.076	-0.204***	0.170***							
8	Professional^d^	0.052	0.034	0.056	0.010	-0.120**	0.091*	-0.245***	–					
9	Technicians and associate professional^d^	-0.197***	-0.095*	0.083	-0.076	0.067	-0.039	-0.141***	-0.151**	–				
10	Clerical support worker^d^	0.324***	-0.197***	-0.031	-0.026	0.236***	-0.205***	-0.357***	-0.383***	-0.220***				
11	Service and sales worker^d^	-0.027	-0.043	-0.128**	0.100*	0.056	-0.020	-0.129**	-0.138**	-0.080	-0.202***	–		
12	Manual worker^d^	-0.111*	0.004	-0.134**	0.042	-0.067	0.043	-0.100*	-0.107*	-0.062	-0.156***	-0.057		
13	JD^e^	-0.029	-0.045	0.063	0.016	-0.067	0.067	0.024	0.227***	-0.004	-0.220***	0.030	-0.020	(0.91)
14	Control	-0.082	0.108*	-0.037	0.019	-0.135**	0.119*	0.213***	-0.019	0.024	-0.130**	-0.071	-0.025	0.026
15	SS^f^	-0.066	-0.024	0.009	-0.003	-0.061	0.064	0.042	0.014	0.011	-0.053	-0.011	-0.016	-0.015
16	CS^g^	-0.004	-0.015	-0.021	0.003	-0.075	0.076	0.044	0.033	-0.035	-0.102*	0.023	0.023	0.017
17	ER^h^	-0.018	-0.002	-0.012	0.020	-0.065	0.068	0.148**	0.007	0.052	-0.112*	-0.036	-0.032	-0.048
18	N^i^	0.059	-0.187***	-0.003	0.021	0.186***	-0.177***	-0.137**	0.008	-0.002	0.023	0.067	0.003	0.139**
19	E^j^	0.009	0.023	-0.061	0.013	-0.148**	0.134**	0.074	0.038	-0.003	-0.101*	-0.025	0.019	0.055
20	C^k^	0.139**	0.217***	-0.010	0.021	-0.042	-0.020	0.182***	-0.024	-0.089*	0.006	-0.081	-0.022	0.093*
21	A^l^	-0.027	0.150**	0.021	-0.022	-0.102*	0.092*	0.105*	-0.004	-0.061	-0.016	-0.046	-0.053	0.018
22	O^m^	-0.103*	0.035	0.070	-0.066	-0.078	0.090*	0.113*	0.053	0.010	-0.124**	-0.065	0.007	0.065
23	WE at baseline^n^	0.021	0.194***	-0.001	0.008	-0.128**	0.102*	0.197***	0.062	0.010	-0.145**	-0.105*	-0.024	0.088*
24	WE at one-year follow-up^n^	0.024	0.274***	0.025	0.023	-0.089*	0.034	0.227***	0.028	-0.001	-0.105*	-0.103*	-0.079	0.073
25	BO at baseline^o^	-0.011	-0.192***	0.005	0.035	0.098*	-0.087	-0.146**	0.025	-0.029	0.019	0.068	0.045	0.288***
26	BO at one-year follow-up^o^	-0.047	-0.196***	0.015	0.011	0.105*	-0.082	-0.128**	0.001	0.022	0.010	0.066	0.039	0.263***
		14	15	16	17	18	19	20	21	22	23	24	25	26
14	Control	(0.81)												
15	SS^f^	0.303***	(0.90)											
16	CS^g^	0.289***	0.748***	(0.88)										
17	ER^h^	0.382***	0.437***	0.369***	(0.87)									
18	N^i^	−0.203***	−0.176***	−0.178***	−0.248***	(0.87)								
19	E^j^	0.224***	0.250***	0.344***	0.247***	−0.226***	(0.88)							
20	C^k^	0.179***	−0.001	0.014	0.128**	−0.245***	−0.010	(0.87)						
21	A^l^	0.187***	0.079	0.122**	0.131**	−0.296***	0.268***	0.357***	(0.87)					
22	O^m^	0.246***	0.152**	0.165***	0.152**	−0.099*	0.477***	0.169***	0.318***	(0.87)				
23	WE at baseline^n^	0.288***	0.316***	0.295***	0.386***	−0.261***	0.300***	0.287***	0.237***	0.271***	(0.98)			
24	WE at one-year follow-up^n^	0.269***	0.258***	0.277***	0.376***	−0.199***	0.200***	0.245***	0.203***	0.210***	0.653***	(0.98)		
25	BO at baseline^o^	−0.363***	−0.322***	−0.321***	−0.434***	0.354***	−0.264***	−0.243***	−0.308***	−0.254***	−0.338***	−0.355***	(0.96)	
26	BO at one-year follow-up^o^	−0.279***	−0.243***	−0.232***	−0.330***	0.354***	−0.222***	−0.260***	−0.287***	−0.200***	−0.335***	−0.387***	0.709***	(0.96)

*p < 0.05; **p < 0.01; ***p < 0.001

^a^Men = 0,Women = 1

^b^High school graduate or lower group is a reference

^c^Divorce or bereavement group is a reference

^d^Others group is a reference

^e^JD = job demands

^f^SS = supervisor support

^g^CS = co-worker support

^h^ER = extrinsic reward

^i^N = neuroticism

^j^E = extraversion

^k^C = conscientiousness

^l^A = agreeableness

^m^O = openness

^n^WE = work engagement

^o^BO = burnout
