# Supplementary material for: Personality is predictive of burnout but not of work engagement: A one-year prospective cohort study
Source: PLoS One. 2026 Jan 7;21(1):e0339258. doi: 10.1371/journal.pone.0339258 (PMC12779045; doi:10.1371/journal.pone.0339258)
Supplement: S1 Appendix — (DOCX) [file pone.0339258.s001.docx]

# Supporting information

**S1 Appendix.** Comparison of demographic characteristics and scale scores between survey completers and dropouts.

|  |  |  | **Completers (*N*= 500)** | | | | |  | **Dropouts (*N*= 1000)** | | | | |  |  |  |
| --- | --- | --- | --- | --- | --- | --- | --- | --- | --- | --- | --- | --- | --- | --- | --- | --- |
| **Demographic characteristics** | | | **Mean (SD)** | |  | ***n* (％)** | |  | **Mean (SD)** | |  | ***n* (％)** | |  | ***p*** | |
|  | Age (years) | | 45.9 | (11.9) |  |  |  |  | 45.6 | (13.4) |  |  |  |  | 0.618 | ^b^ |
|  | Gender | |  |  |  |  |  |  |  |  |  |  |  |  |  |  |
|  |  | Men |  |  |  | 299 | (59.8) |  |  |  |  | 458 | (45.8) |  | <0.001 | ^c^ |
|  |  | Women |  |  |  | 201 | (40.2) |  |  |  |  | 542 | (54.2) |  |  |  |
|  | Education | |  |  |  |  |  |  |  |  |  |  |  |  |  |  |
|  |  | University/graduate school graduate |  |  |  | 320 | (64.0) |  |  |  |  | 553 | (55.3) |  | 0.006 | ^c^ |
|  |  | Vocational school/college graduate |  |  |  | 102 | (20.4) |  |  |  |  | 252 | (25.2) |  |  |  |
|  |  | High school graduate or lower |  |  |  | 78 | (15.6) |  |  |  |  | 195 | (19.5) |  |  |  |
|  | Marital status | |  |  |  |  |  |  |  |  |  |  |  |  |  |  |
|  |  | Unmarried |  |  |  | 171 | (34.2) |  |  |  |  | 317 | (31.7) |  | 0.064 | ^c^ |
|  |  | Married |  |  |  | 283 | (56.6) |  |  |  |  | 550 | (55.0) |  |  |  |
|  |  | Divorce or bereavement |  |  |  | 46 | (9.2) |  |  |  |  | 133 | (13.3) |  |  |  |
|  | Occupations | |  |  |  |  |  |  |  |  |  |  |  |  |  |  |
|  |  | Manager |  |  |  | 93 | (18.6) |  |  |  |  | 144 | (14.4) |  | 0.004 | ^c^ |
|  |  | Professional |  |  |  | 104 | (20.8) |  |  |  |  | 226 | (22.6) |  |  |  |
|  |  | Technicians and associate professional |  |  |  | 40 | (8.0) |  |  |  |  | 65 | (6.5) |  |  |  |
|  |  | Clerical support worker |  |  |  | 179 | (35.8) |  |  |  |  | 310 | (31.0) |  |  |  |
|  |  | Service and sales worker |  |  |  | 34 | (6.8) |  |  |  |  | 99 | (9.9) |  |  |  |
|  |  | Manual worker^a^ |  |  |  | 21 | (4.2) |  |  |  |  | 57 | (5.7) |  |  |  |
|  |  | Others |  |  |  | 29 | (5.8) |  |  |  |  | 99 | (9.9) |  |  |  |
| **Scale scores** | | | **Mean (SD)** | |  | ***n* (％)** | |  | **Mean (SD)** | |  | ***n* (％)** | |  |  |  |
|  | Five-factor model | |  |  |  |  |  |  |  |  |  |  |  |  |  |  |
|  |  | Neuroticism | 16.0 | (3.7) |  |  |  |  | 16.1 | (4.0) |  |  |  |  | 0.501 | ^b^ |
|  |  | Extraversion | 15.2 | (3.7) |  |  |  |  | 15.4 | (3.8) |  |  |  |  | 0.226 | ^b^ |
|  |  | Conscientiousness | 22.5 | (4.3) |  |  |  |  | 22.4 | (4.6) |  |  |  |  | 0.696 | ^b^ |
|  |  | Agreeableness | 19.2 | (3.6) |  |  |  |  | 19.3 | (3.6) |  |  |  |  | 0.345 | ^b^ |
|  |  | Openness | 18.3 | (3.8) |  |  |  |  | 18.2 | (4.0) |  |  |  |  | 0.555 | ^b^ |
|  | Job demands | | 16.3 | (3.9) |  |  |  |  | 16.5 | (4.1) |  |  |  |  | 0.340 | ^b^ |
|  | Job resources | |  |  |  |  |  |  |  |  |  |  |  |  |  |  |
|  |  | Control | 8.0 | (2.1) |  |  |  |  | 8.0 | (2.2) |  |  |  |  | 0.770 | ^b^ |
|  |  | Supervisor support | 7.4 | (2.3) |  |  |  |  | 7.4 | (2.5) |  |  |  |  | 0.595 | ^b^ |
|  |  | Co-worker support | 7.8 | (2.2) |  |  |  |  | 7.8 | (2.4) |  |  |  |  | 0.620 | ^b^ |
|  |  | Extrinsic reward | 18.0 | (3.5) |  |  |  |  | 17.7 | (3.4) |  |  |  |  | 0.117 | ^b^ |
|  | Work engagement at baseline | | 21.5 | (12.3) |  |  |  |  | 22.4 | (12.5) |  |  |  |  | 0.146 | ^b^ |
|  | Burnout at baseline | | 31.7 | (8.9) |  |  |  |  | 32.0 | (8.9) |  |  |  |  | 0.580 | ^b^ |

^a^Manual worker include craft and related trades worker, plant and machine operator and assembler, and elementary occupation.

^b^t-test

^c^chi-square test
