# Supplementary material for: Personality is predictive of burnout but not of work engagement: A one-year prospective cohort study
Source: PLoS One. 2026 Jan 7;21(1):e0339258. doi: 10.1371/journal.pone.0339258 (PMC12779045; doi:10.1371/journal.pone.0339258)
Supplement: S2 Appendix — Note. Weighted by stabilized inverse probability weights to adjust for potential attrition bias. Results correspond to the final step (Step 5) of the main analysis (Table 3). aMan = 0, Woman = 1. (DOCX) [file pone.0339258.s002.docx]

# Supporting information

**S2 Appendix.** Association of work engagement at baseline, demographic characteristics, job demands, job resources, and personality with work engagement at one-year follow-up: hierarchical multiple regression analysis weighted by stabilized inverse probability weights (*N* = 500).

|  | | ***β*** | | **95%CI** | | |
| --- | --- | --- | --- | --- | --- | --- |
| Work engagement at baseline | | 0.527 |  | 0.428 | – | 0.625 |
| Demographic characteristics | |  |  |  |  |  |
|  | Gender^a^ | 0.021 |  | -0.136 | – | 0.177 |
|  | Age (years) | 0.194 |  | 0.109 | – | 0.278 |
|  | Education (reference = high school graduate or lower) |  |  |  |  |  |
|  | University/graduate school graduate | 0.176 |  | -0.024 | – | 0.376 |
|  | Vocational school/college graduate | 0.171 |  | -0.048 | – | 0.389 |
|  | Marital status (reference = divorce or bereavement) |  |  |  |  |  |
|  | Unmarried | -0.061 |  | -0.300 | – | 0.177 |
|  | Married | -0.257 |  | -0.476 | – | -0.038 |
|  | Occupation (reference = others) |  |  |  |  |  |
|  | Manager | 0.057 |  | -0.348 | – | 0.461 |
|  | Professional | -0.094 |  | -0.486 | – | 0.298 |
|  | Technicians and associate professional | 0.021 |  | -0.418 | – | 0.461 |
|  | Clerical support worker | 0.012 |  | -0.371 | – | 0.396 |
|  | Service and sales worker | -0.197 |  | -0.603 | – | 0.210 |
|  | Manual worker | -0.255 |  | -0.737 | – | 0.226 |
| Job demands | | 0.053 |  | -0.027 | – | 0.133 |
| Job resources | |  |  |  |  |  |
|  | Control | 0.022 |  | -0.069 | – | 0.113 |
|  | Supervisor support | -0.071 |  | -0.172 | – | 0.030 |
|  | Co-worker support | 0.139 |  | 0.030 | – | 0.248 |
|  | Extrinsic reward | 0.143 |  | 0.058 | – | 0.227 |
| Personality | |  |  |  |  |  |
|  | Neuroticism | -0.018 |  | -0.109 | – | 0.072 |
|  | Extraversion | -0.045 |  | -0.131 | – | 0.041 |
|  | Conscientiousness | -0.015 |  | -0.094 | – | 0.063 |
|  | Agreeableness | 0.011 |  | -0.053 | – | 0.076 |
|  | Openness | 0.025 |  | -0.064 | – | 0.114 |

*Note.* Weighted by stabilized inverse probability weights to adjust for potential attrition bias. Results correspond to the final step (Step 5) of the main analysis (Table 3).

^a^Man = 0, Woman = 1
