# Supplementary material for: Personality is predictive of burnout but not of work engagement: A one-year prospective cohort study
Source: PLoS One. 2026 Jan 7;21(1):e0339258. doi: 10.1371/journal.pone.0339258 (PMC12779045; doi:10.1371/journal.pone.0339258)
Supplement: S3 Appendix — Note. Weighted by stabilized inverse probability weights to adjust for potential attrition bias. Results correspond to the final step (Step 5) of the main analysis (Table 4). aMan = 0, Woman = 1. (DOCX) [file pone.0339258.s003.docx]

# Supporting information

**S3 Appendix.** Association of burnout at baseline, demographic characteristics, job demands, job resources, and personality with burnout at one-year follow-up: hierarchical multiple regression analysis weighted by stabilized inverse probability weights (*N* = 500).

|  | | ***β*** | | **95%CI** | | |
| --- | --- | --- | --- | --- | --- | --- |
| Burnout at baseline | | 0.586 |  | 0.491 | – | 0.681 |
| Demographic characteristics | |  |  |  |  |  |
|  | Gender^a^ | -0.084 |  | -0.226 | – | 0.058 |
|  | Age (years) | -0.031 |  | -0.108 | – | 0.046 |
|  | Education (reference = high school graduate or lower) |  |  |  |  |  |
|  | University/graduate school graduate | -0.049 |  | -0.234 | – | 0.136 |
|  | Vocational school/college graduate | -0.022 |  | -0.210 | – | 0.166 |
|  | Marital status (reference = divorce or bereavement) |  |  |  |  |  |
|  | Unmarried | 0.011 |  | -0.198 | – | 0.219 |
|  | Married | -0.011 |  | -0.196 | – | 0.174 |
|  | Occupation (reference = others) |  |  |  |  |  |
|  | Manager | 0.074 |  | -0.235 | – | 0.383 |
|  | Professional | 0.033 |  | -0.254 | – | 0.320 |
|  | Technicians and associate professional | 0.149 |  | -0.249 | – | 0.546 |
|  | Clerical support worker | 0.076 |  | -0.224 | – | 0.376 |
|  | Service and sales worker | 0.116 |  | -0.230 | – | 0.463 |
|  | Manual worker | 0.046 |  | -0.349 | – | 0.441 |
| Job demands | | 0.095 |  | 0.022 | – | 0.168 |
| Job resources | |  |  |  |  |  |
|  | Control | -0.034 |  | -0.108 | – | 0.041 |
|  | Supervisor support | -0.035 |  | -0.141 | – | 0.070 |
|  | Co-worker support | 0.024 |  | -0.083 | – | 0.130 |
|  | Extrinsic reward | -0.009 |  | -0.084 | – | 0.067 |
| Personality | |  |  |  |  |  |
|  | Neuroticism | 0.089 |  | 0.017 | – | 0.162 |
|  | Extraversion | -0.033 |  | -0.112 | – | 0.046 |
|  | Conscientiousness | -0.074 |  | -0.147 | – | -0.0003 |
|  | Agreeableness | -0.036 |  | -0.112 | – | 0.040 |
|  | Openness | 0.012 |  | -0.059 | – | 0.082 |

*Note.* Weighted by stabilized inverse probability weights to adjust for potential attrition bias. Results correspond to the final step (Step 5) of the main analysis (Table 4).

^a^Man = 0, Woman = 1
